# Transformative experience and informed consent to psychedelic-assisted psychotherapy

**DOI:** 10.3389/fpsyg.2023.1108333

**Published:** 2023-05-26

**Authors:** Edward Jacobs

**Affiliations:** ^1^Department of Psychiatry, Medical Sciences Division, University of Oxford, Oxford, United Kingdom; ^2^Wellcome Centre for Ethics and Humanities, Nuffield Department of Population Health, Medical Sciences Division, University of Oxford, Oxford, United Kingdom

**Keywords:** informed consent, transformative experience, psychedelic-assisted psychotherapy, value change, consent, psychedelic, psychedelic ethics

## Abstract

Just as psychedelic-assisted psychotherapy (PAP) represents a clinical innovation that may need to be accommodated with corresponding theoretical and methodological innovations, there is growing awareness that the tools, normative frameworks, and standard practices of our clinical ethics may also need to be adapted, renewed, or replaced to accommodate its unusual features. Drawing on L. A. Paul's work on “Transformative Experience,” I argue that the acute and long-term effects that are repeatedly reported following the administration of psychedelic drugs, including in clinical contexts, are epistemically inaccessible at the point of deciding to take them. By virtue of both the so-called “mystical” experiences that frequently arise during PAP, and the long-term shifts to outlooks, values, and priorities that can follow treatment, the processes of decision-making that are normatively expected of patients run aground. If this framing is correct, then prospective patients cannot meet the requirement of understanding that is one of the principal analytic components of informed consent. The role of understanding in supporting two functions of informed consent—avoiding unauthorized trespass against patients and supporting values-aligned decision-making—is explored, and I argue that, while the normative standard for the first function may be met by extant suggestions for enhancing the consenting process for PAP, the latter function remains unattainable. In light of this, the consequences for the ethical preparation of prospective patients are considered.

## 1. Introduction

The anticipated (re-)introduction of classical psychedelics into psychiatry (chiefly among them, psilocybin and LSD) is likely to bring with it a number of challenges to standard models of practice. This has already proven the case as psilocybin works its way through the drug licensing process, wherein its particularly obvious acute subjective effects, psychotherapeutic components, and the challenge for effective blinding that these bring impose a complication in meeting the evidentiary gold standard of the double-blind, placebo-controlled clinical trial (Muthukumaraswamy et al., 2021; Schenberg, 2021; Aday et al., 2022). Similarly, there is growing awareness that the atypical features of psychedelic-assisted psychotherapy (PAP), the hybrid pharmacotherapy-psychotherapy modality of psychedelic medicine, may demand the adaptation, renewal, or replacement of the tools, normative frameworks, and standard practices of our clinical ethics (Brennan et al., 2021; Smith and Sisti, 2021; Smith and Appelbaum, 2022).

The current study is an exploration of one apparent tension between the familiar tools and practices of medical ethics and the atypical features of PAP. I argue that informed consent, as standardly conceived as a legitimacy requirement for medical intervention (Eyal, 2018), may not be possible before undertaking PAP. In brief, this is because informed consent demands that a prospective patient be presented with, and understands, an account of the intended treatment that is not materially incomplete, such that she is equipped to autonomously authorize a course of action while understanding whether, and how, it aligns with her values—i.e., to choose a treatment that is *right for her*. Using the framework of philosopher L. A. Paul's “transformative experience” (Paul, 2014), I propose that prospective patients cannot undertake a rational reflection of whether PAP is right for them, given that materially relevant facts about the treatment remain epistemically inaccessible to patients before treatment has begun. This is because of two features of the psychedelic experience: first, the epistemically transformative nature of the acute drug effects, which can be of so radically different a nature to previous experience as to be fully comprehensible only by experiencing it. More crucially, clinical evidence supports the thesis that PAP can be of a personally transformative nature: repeatedly recorded downstream changes following psychedelic use, including rapid and robust shifts to values, personality, beliefs, and behavior (Griffiths et al., 2008, 2011; MacLean et al., 2011; Timmermann et al., 2021; Nayak et al., 2023), have the potential to change the very beliefs, value set, and core preferences against which people make decisions. As such, there are grounds to believe that the idealized conception of meaningfully informed consent, which we standardly seek before beginning treatment with a competent patient, could be an inappropriate tool for legitimizing a course of PAP.

## 2. Consent and informed consent

The act of consenting is central to our interactions in many spheres—most prominently sexual, commercial, and medical, and its importance is so widely accepted that its function might easily go unarticulated. At its center across all these contexts is a recognition of the need to respect autonomy. While a full normative analysis of the varying conceptions of autonomy is neither possible nor necessary here (see Christman, 1988, or Taylor, 2005, for an overview), it suffices for current purposes to point to the intuitive core that they share—the value of the freedom to be authors of our own lives, choosing what we think best for us in matters of importance to us, and remaining sovereign over our own bodies (Beauchamp and Childress, 2019). Because of this, we consider people to have, at default, a “perimeter of rights” of non-interference against their person, property, or lives (Dougherty, 2020, p. 138). However, since we, at times, want to relax that perimeter to interact meaningfully and valuably with others, the “autonomous authorization” (Faden and Beauchamp, 1986) of valid consent to a specific action serves to toggle that protection on and off, albeit in a precisely delineated manner,^1^ that is, consent functions to *waive* important ethical requirements in limited ways and particular contexts so as to license actions that would otherwise be ethically or legally unacceptable (Manson and O'Neill, 2007, ch. 4). Consent turns “a rape into love-making, a kidnapping into a Sunday drive, a battery into a football tackle, a theft into a gift, and a trespass into a dinner party” (Hurd, 2004). Hurd (1996) goes so far as to ascribe to consent as a “moral magic.”

Standardly, an *autonomous authorization* is viewed as comprising three components: It must be intentional, voluntary, and made by an actor with sufficient understanding of what is being authorized (Beauchamp, 2009), but note that the thresholds for voluntariness and understanding, and thereby for valid and morally forceful consent, can vary across contexts. In an oligopsony market, in which there are many suppliers but few buyers (in Australia, two supermarkets control some 70% of the national food market), a buyer may threaten not to renew a supply contract unless a significant reduction in price is agreed: Such an agreement may not be wholly voluntary but remains valid. The act of ordering a bottle of champagne at a club is seen as tokening a valid consent to being charged for it, even where a price is never mentioned. A drunk first-time gambler can bet his life savings on a spin of the roulette wheel, consenting to have his stake taken if the ball lands on red instead of black. In these, and many other mundane interactions, we recognize the moral power of that consent even when the acts in question are potentially damaging to the consenting parties' interests or wellbeing.

But the bar is set much higher in clinical research and healthcare (Eyal, 2018)—here alone, we talk of “informed consent” rather than “consent.” The common function of consent—safeguarding autonomy—remains, and with it, a toggling off of important rights, most obviously to bodily integrity. To add to Hurd's list, consent can turn a battery into a life-saving surgery. In doing so, the consent-taking process also serves an important institutional role in protecting physicians from litigation. Requiring consent to be *informed* also serves as an additional level of defense for patients in recognition of a particular vulnerability: Across the physician–patient relationship, wherein highly important rights are to be waived, there is a significant asymmetry of information. The point of drawing this distinction is to highlight that different norms govern the practice of giving and accepting consent across different relationships and contexts. Although the variability in these norms does not undermine the validity of consent in any given context, we ought to tread carefully in eliding norms across situations.

While safeguarding patient autonomy is a key function of informed consent in healthcare, contemporary discussion signals that this is not the only role we want it to perform (Dickert et al., 2017). Among those diverse goals, that which is of interest here, “[o]ne of the most widely accepted goals for informed consent[,] is to promote more informed healthcare decisions in accordance with patients' values” (Berg et al., 2001, ch. 14). It is not enough that a physician has decided that an intervention is medically the best course of action but also that, for the patient themselves, “[a]dvantages and disadvantages have to be understood and weighed rationally…patients have to be made aware of both the informative and emotional content of a decision” (Dsubanko-Obermayr and Baumann, 1998). Supporting decision-making that allows patients the opportunity to choose treatments in accordance with their values is driven by wanting their choices to reflect who they are and what they care about, or, in everyday terms, if a treatment is *right for them*.^2^^,^^3^ Something has gone wrong in the informed consent process if a patient who ardently wants to bear children unwittingly accepts a treatment with a high risk of resulting in sterility, or a Jehovah's Witness agrees to an intervention that unbeknown to her involves a blood transfusion, where other options are available.

As such, a secondary function of informed consent, and a secondary obligation imposed on clinicians, is to promote value-aligned decision-making, where this is understood as supporting patients to “weigh the pros and cons of the alternative choices at hand and choose the option that most aligns with their values, needs, and belief” (Rogers and Johnson, 2021). Doing so secures or enhances the *instrumental* value of the autonomy of patients, i.e., that of “enabling persons to act to attempt to satisfy their own desires and secure their own goals” (Taylor, 2010, p. 141). This function—helping patients to avoid mistakes in their decision-making process about what is *right for them—*might stem from a generalized clinical duty of *beneficence*, or more specifically the physician's role as a fiduciary toward the patient (Joffe and Truog, 2010).^4^

To draw out why PAP presents a challenge for informed consent, it is useful to outline the analytic components of informed consent that are frequently appealed to across legal, medical, and philosophical contexts, namely, (1) competence, (2) disclosure, (3) understanding, (4) voluntariness, and (5) consent.

The pertinent component when considering transformative experiences is *understanding*. For a number of scholars, understanding takes a lexical priority over *disclosure*: Disclosure of the nature and details of a prospective intervention, in the absence of understanding, does not seem to be sufficient for informed consent—“[p]lainly, comprehension is essential for truly informed consent, for the act of disclosure would otherwise be pointless” (Capron, 2008, p. 625)—while disclosure may not be necessary where the patient is already in a position of understanding. The relevant information may be naturally understood in a context where a physician applies a bandage to a profusely bleeding wound (Pugh, 2020, p. 163), or, for more involved interventions, where the patient in question is a colleague in the same medical specialty as the treating physician (Faden and Beauchamp, 1986, p. 276). The *understanding* component bears upon both the *autonomous authorization* and *supporting value-aligned decision-making* functions of informed consent. Because of the informational asymmetry often at play in medical contexts, there is a high risk of both *referential opacity* and *failure to grasp the consequences*. This is chiefly because consent is a propositional attitude; consent to a treatment described one way does not necessarily entail consent to the same treatment described differently: A description couched less euphemistically, or with an emotionally richer articulation of *what it is like* to experience its side-effects (O'Neill, 2002, p. 42–44), might yield different results.

For consent to count as *autonomous authorization* in a medical context, the threshold for understanding is higher than that we seek from the club patron ordering champagne in ignorance about its price. Where a doctor fails to take steps to ensure that her patient *understands* the nature and implications of a particular treatment, she thereby deprives them of the opportunity to reflect on how the descriptive facts concerning the treatment align with their own values and preferred ways of living—i.e., whether the treatment is right *for them*. For fulfilling either function of informed consent, it is a contested matter as to just how much information, or precisely what information, must be understood. Clearly, informed consent cannot be secured if *nothing* of the proposed intervention is understood, as when there is no common language between doctor and patient, and no interpreter is present. Nor must *everything* be understood: understanding everything about an intervention would require a high degree of medical expertise on the part of the patient, and any workable account of informed consent cannot require that patients themselves be medical experts. In both philosophical and legal discussions of informed consent, an appeal is given to the concept of *materiality*—information concerning the procedure that is *material* to the patient's decision must be understood. Whether a 16-gauge or 18-gauge needle is used in venipuncture exemplifies a non-material aspect of the treatment; however, a treatment's risk of causing infertility is a clear example of a material aspect, impinging as it does on the values and important ends of a patient. There is a vagueness to this distinction, and while different conceptions have sought to articulate clear bright lines to define the threshold of materiality in any given circumstance,^5^ for current purposes, we can remain agnostic here: As discussed below, the kinds of changes that have been recorded following PAP will count as material to treatment decisions on any plausible account.

## 3. Transformative experience and choosing

A helpful framework through which to understand the challenge to informed consent from PAP is philosopher L. A. Paul's “transformative experience” (Paul, 2014). Paul demarcates a class of experiences that have two important features that impose a challenge on our decision-making about *what is right for us*. The first feature is that they are *epistemically transformative*—because they are so unlike any previous experience, a person cannot imagine what the experience will be like for them except by undergoing it. In addition, they are *personally transformative*—the act of undergoing the experience changes someone's perspectives, priorities, or core values such that, in a sense, the kind of person they are changes. Paul offers a dramatic example to highlight these features—being presented with a genuine opportunity to become a vampire (Paul, 2014, p. 2). As Paul astutely observes, if you were to take up this offer, “life will be completely different.” But the nature of this difference, as it is experienced by you, is epistemically inaccessible at the point of choosing. This is because, never having been a vampire before, “you cannot compare the character of the lived experience of what it is like to be you… to the character of the lived experience of what it is like to be a vampire” (p. 4). Moreover, though you might prioritize sunbathing as a prized form of relaxation now, or a vegan diet as the pinnacle of nutrition and a moral necessity, these ideas become positively repugnant after *the change*.

Paul draws attention to the reality that we face similar, if less fantastical, choices throughout life—typically “central, life-defining choices” (p. 94) which involve transformative experiences, the lived reality of which is epistemically inaccessible at the point of choosing them, and which can change both *us*, and *what we care about*, in ways that we cannot anticipate, which thereby “limits our ability to make informed, rational, and authentic plans” (Sebo and Paul, 2019, p. 1).

The paradigmatic case that Paul deploys to explain the character of transformative experience is that of having a child. Having your first child is *epistemically transformative*, in that it allows an individual to grasp new knowledge (i.e., what it is like to have a child), that is epistemically accessible *only* through having your first child: Parents are known to remark that no amount of babysitting nieces and nephews, nappy-changing and all, truly informs a person as to *what it is really like to have your own child*.^6^ More crucially, the experience of having a child is *personally transformative*: By having and raising a child, *you* can be transformed in a fundamental, personal way—through the updating or development of your core personal values, beliefs, and practices: “Your preferences will change. The way you live your life will change. What and who you care about will change” (Paul, 2014, p. 80–81). Such experiences are significant in that they “function as crossroads in your path toward self-realization” (Paul, 2014, p. 17). An experience that changes your point of view enough to revise your core values and preferences, or how you see yourself in the world, has consequences that can reverberate through the rest of your life.

Transformative experiences are ethically significant because they demonstrate that our preferred conception of how we make important decisions in our life is lacking in some way. We cannot decide whether to have a child by rationally weighing the pros and cons of having a child vs. remaining childless, choosing outcomes that align with our values and preferences, evaluating “each possible act and its experiential outcomes by imagining or running a mental simulation of what it would be like” (Paul, 2014, p. 26). Firstly because “one cannot determine the value of what it is like to have one's own child before actually having her” (Paul, 2015, p. 11). But it is not only that there is a deep ignorance about what it is *really* like to have *your* child (they're not all alike, and who your child is makes a big difference to your experience of parenthood). Additionally, anyone considering having children must grapple with their ignorance about what it would really be like to undergo a fundamental shift in their values, preferences, and worldview upon becoming a parent. The practice of making transformative decisions through rational decision theory, estimating the subjective value *for you* of each option, is thereby doubly frustrating: you don't know what the experience will be like, and you don't know which values and preferences you will have in the wake of the experience, and what it is like to have them, by which to judge its value.^7^ These “central, life-defining choices” intractably involve a leap of faith—a commitment to discovering the unknown. This discovery is not simply of what it will be like to have some novel experience, but also, and more pressingly, the discovery of *who you will become*, should the experience give you attitudes, a worldview, and values that are at the point of the choice alien to you. Salvaging a rational weighing of options by appeal to third-person accounts, or social scientific data, is of limited use in such circumstances, if we value *making our own choice*. Paul (2014) rejects this option on existentialist grounds, going so far as to call it “disastrous” (p. 87), doing “great violence to our ordinary way of thinking about deliberation” (p. 128). Someone who chooses to have a child against her own desires on the basis of such data, “in effect, turns her decision over to the experts and eliminates consideration of her personal references, [and] seems to be giving up her autonomy for the sake of rationality” (Paul and Bloom, 2015). There is significant merit to this view: Fundamentally we want our “central, life-defining choices” to ultimately be *our* choices, and, thus, electing to undergo a transformative experience on the basis of third-person or population data appears like a misstep, if not an outright evacuation of responsibility. A 0% regret rate among vampires is not as reassuring as the number alone suggests—“do you want to become a vampire, live a vampire life, have vampire values?” is a question not just about which option maximizes your subjective expected value but about deciding *who you want to become*. Or suppose that, wanting to remain childless, you are presented with data that supports the thesis that having children confers higher life satisfaction, or greater meaning, or more of any other desirable end. The transformational experience of having a child remains open to you, but to be swayed by this data appears to be countermanding your own preferences in a sphere in which your own preferences are of significant importance.

## 4. Does psychedelic-assisted psychotherapy involve transformative experience?

To begin with, the usual: the experience is so fantastic in both its novelty and its power as to beggar all possibility of adequate depiction through words. The most that can be hoped for by way of description is an approximation, and only those who have had the drug can know how far removed from actuality the approximation must be.

-Scholar of religions Huston Smith, quoted in Ulrich, 2018.^8^

Alongside many such poetic tributes to the psychedelic experience, there is empirical evidence that supports the interpretation of PAP, and the changes that have been reported to follow it, as “transformative” in both Paul's *epistemic* and *personal* sense. I say “supports,” rather than “demonstrates,” because there are no objective criteria that can be appealed to for demarcating transformation in either sense. Clinically relevant doses of psychedelics frequently involve “peak” or “mystical” experiences, reported repeatedly in the context of clinical and experimental psychedelic use (Pahnke, 1967; Garcia-Romeu et al., 2014; Griffiths et al., 2016). These are characterized in part by a sense of sacredness, strongly felt positive mood, transcendence of space and time, a noetic quality—the subjective feeling of accessing knowledge or revelation unmediated by usual sources of validation or evidence—and ineffability. Alongside these is *ego dissolution*, the disintegration of the perceived boundary between the self and the external world, and occasionally quasi-synaesthetic^9^ (Studerus et al., 2012) perceptions. These acute drug effects, and the other phenomenological aspects of a psychedelic trip (Preller and Vollenweider, 2018), are exemplary candidates for epistemic inaccessibility, with psychedelic “trips” representing a radical departure from previous experience.^10^ This framing is consistent with What fMRI evidence is available which examines the psychedelic state—namely, that it is driven by significantly altered patterns of cortical activity. At a first gloss, the changes are characterized by the disintegration and desegregation of typically stable brain networks, meaning that, at a neural level, information processing takes place in ways that are markedly different from what has been experienced before (Carhart-Harris, 2019). For example, the functional connectivity of the primary visual cortex under LSD is greatly expanded, correlating with simple hallucinations and complex imagery, suggesting that considerably more parts of the brain contribute to visual processing during acute drug effects than in normal conditions (Carhart-Harris et al., 2016).

The potentially *personally* transformative nature of the psychedelic experience manifests in a range of ways. Findings in healthy and clinical populations include increases in the personality domain of openness sustained in the weeks and months after drug sessions (MacLean et al., 2011; Lebedev et al., 2016; Erritzoe et al., 2018). Openness is typified by aesthetic sensitivity, attentiveness to inner feelings, and intellectual curiosity (Costa and McCrae, 1992), where each characteristic that is described as developing in qualitative reports by patients (Watts et al., 2017; Noorani et al., 2018). Shifts in attitudes toward life and the self are also frequently reported (Studerus et al., 2011; Gasser et al., 2015; Ross et al., 2016; Belser et al., 2017; Johnson et al., 2017; Schmid and Liechti, 2018), although the magnitude of these effects is not consistently pronounced across research centers (Nicholas et al., 2018; McCulloch et al., 2022). For some patients, PAP has resulted in renewed confidence and determination to pursue long-valued goals that were nonetheless orthogonal to the target condition (Swift et al., 2017, p. 20). In others, the treatment led to the revision of life priorities and lifestyle preferences (Belser et al., 2017; Forstmann and Sagioglou, 2017), the discovery of new values and preferences, and the adoption of new habits and activities (Watts et al., 2017). Persisting life changes following PAP are attested to as much as 4.5 years following treatment (Agin-Liebes et al., 2020). Since much of the data regarding these changes derives from using psilocybin to treat existential anxiety secondary to a cancer diagnosis, or treatment-resistant depression, one possibility is that these changes are simply natural consequences of remission in these serious conditions and might equally come about following *any* successful treatment. However, this is not plausible for all such shifts—such as the sustained adoption of a vegetarian diet (Watts et al., 2017, p. 559), while changed relationships with loved ones and alterations to long-standing habits, attitudes, and priorities have been recorded after PAP for tobacco cessation (Noorani et al., 2018). In the latter study, participants were smokers averaging 18 cigarettes a day, decades of smoking, and seven previous quit attempts. While changes were, in some cases, noted even in the absence of treatment success, where treatment resulted in tobacco cessation, it was “often reported as one of the *least* important effects of the study for participants in retrospect” (Noorani et al., 2018, p. 763).

Not least because psychedelic experiences are not *always* transformative in these ways—those subject to psychedelic administration can experience no such change, deteriorations (Studerus et al., 2011), or instead a reinforcing of extant worldviews (Pace and Devenot, 2021)—the precise nature and cause of these changes, and how patients come to understand them, are still unclear, remaining an important avenue for further study. One psychological model to account for the clinical changes following PAP, which might also be used to explain the non-clinical changes described here, dovetails neatly with the concept of transformative experience. Hendricks (2018) proposes that the profound awe that characterizes psychedelic-induced mystical experiences—a response to a stimulus perceived as far larger than the self, or standard experiential anchors of comparison—demands a cognitive accommodation, “the need to adjust mental structures so as to integrate [the experience].” Brouwer and Carhart-Harris (2021) operationalize a construct overlapping with transformative experiences, including but not limited to psychedelic experiences, in neurobiological terms as a “Pivotal Mental States.” These states (rather than the potentially transformative outcomes they can induce) are defined by Brouwer and Carhart-Harris as “*transient, intense hyper-plastic mind and brain states, with exceptional potential for mediating transformation*,” suggesting more objective criteria of “(a) *elevated cortical plasticity*, (b) *an enhanced rate of associative learning*, and (c) *a unique capacity to mediate psychological transformation*” (p. 320). As well as the advantage of generalizability across other pivotal mental states, this account benefits from an explicit valence agnosticism regarding such transformations: A hyper-plastic state that enhances the likelihood of major psychological change is not good *per se*, as the examples collated above might suggest, but rather the broad valence and precise nature of ensuing changes is dependent on contextual factors.

This valence agnosticism is of particular clinical importance when considering the role of transformative experience in PAP, not only because it underlines the need for research on how to shape contextual factors to minimize negatively experienced transformations but also because the reality of the potential for negative outcomes needs to be made apparent to prospective patients, whose prior perceptions of PAP may be unduly informed by science communications and a wider media that has for some years been beholden to a hype bubble of inflated expectations (Yaden et al., 2022). The impact of this hype is not trivial—suicidal behavior was recorded among three participants of one trial of PAP for treatment-resistant depression, and a demoralization effect, the affective response that *not even this much reported cure-all* can relieve my symptoms, might plausibly contribute to hopelessness (Gukasyan, 2023).^11^ As with the clinical effects of psychedelics, the broader effects of interest here are far from guaranteed. One early pooled analysis of psilocybin studies found that, while 18% of participants reported positively-assessed changes in values and 25% positively-assessed changes in relationships with other people, about one-quarter as many reported that their values (5%) or relationships with others (7%) changed for the worse (Studerus et al., 2011). If and when PAP becomes a mainstream medical intervention, such negative changes could be a reality for significant numbers of patients.

Whatever the mechanism supporting these changes, their character, coupled with the repeated finding from one research center that psychedelic experiences are often counted among the most personally meaningful and spiritually significant experiences in a person's life—on a par with the birth of a first child or death of a parent (Griffiths et al., 2006; Johnson et al., 2017; Schmid and Liechti, 2018)—it should be taken as a serious possibility that the long-term effects of PAP are personally transformative in the sense described by Paul (2014) that is such a challenge for informed consent.

## 5. Do they know what they are getting themselves in for? Uncertain outcomes and transformative experiences across medicine

Are transformative experiences a particular problem for informed consent in medicine, when uncertainty about outcomes is part and parcel of the practice of medicine? In medicine (and elsewhere), the outcomes of our decisions are not guaranteed. This ignorance about the future is an inevitable part of life, and part of what living an autonomous life *is*, is deciding how to act in the face of uncertainty. But typically when choosing between medical treatment plans, a patient who is provided with the probabilities of treatment success and of side-effect risks for various options is nonetheless still *informed* in a materially significant way that does not preclude rational reflection on what is right for them: By knowing about the character, severity, and likelihood of possible outcomes, and reflecting on the personal impact of these potential outcomes, patients can model, at least roughly, the expected subjective value of each choice. Within this commonplace gray area of uncertainty, the physician provides what expertise they can, and the patient makes their best guess on the basis of this information.

The challenge from transformative experiences is a distinct one: It is not garden-variety uncertainty, in which, at the moment of choice, the patient cannot know which of the potential outcomes will come to pass and must in some sense roll a dice and hope for the best. Rather, it is that the rational, value-oriented processes of decision-making under uncertainty themselves run aground. For epistemically transformative experiences, without being able to model *what it will be like* for one or more of the potential outcomes of a choice to come to pass, she cannot assign that choice an expected subjective value. This is not a *best guess*—it is just a guess. For potentially personally transformative experiences, in which a choice can bring about deep changes to your values and perspectives: “the edifice of our choice model stands on shifting sands: in virtue of having the transformative experience we've chosen, we change what we care about… it means that, if you choose to have the experience, it will change who you are. This affects the way we understand how the decision ‘turns out”' (Paul, 2019, p. 358–359). Deciding whether to have a child, to join the military or a monastery, or to undergo any transformative experience, is not simply about what will maximize your subjective expected value, but rather about whether you are willing to risk becoming someone different; however, PAP does not stand alone as the only treatment with transformative features known to medicine. Given that epistemically transformative experiences are as accessible as trying a new fruit for the first time, it would be odd for them *not* to arise in healthcare settings. But *any* psychoactive medicine to which a patient is naive represents an epistemically transformative experience, as well as many instances of pain that are largely medical procedure-specific, among which are uteroscopies, bone marrow donations, and dental implants. Inasmuch as PAP provides a novel challenge for informed consent, it is because of their potentially *personally* transformative nature.

While personally transformative choices can be found elsewhere in medicine, note that they characteristically take a different form which is distinct when considering informed consent. Challenging pregnancies or deliveries, or some cases of pediatric neurosurgical disease (Shlobin et al., 2022), bring to light the reality that there are some medical contexts in which all roads lead to transformative experience—for example the choice between living with the death of a child or with a permanently disabled child. Any available treatment option (or electing not to treat) is liable to profoundly change how you see yourself, how you see the world, or what is important to you. Suppose that the lesson you took away from this article (*pace* my suggestions below) was that, because transformative experience makes truly informed consent impossible, any potentially transformative procedures would become so ethically hazardous as to be impermissible for clinicians to perform. This would not mean that the ethical hazard of transformative experience is avoided since medical inaction can result in transformation as much as action.

There are some scenarios, though, where only one medical choice leads to personal transformation. Paul's exemplar transformative experience, having children, is at least sometimes accommodated within the medical realm, as when parents seek support in conceiving through *in vitro* fertilization and other fertility treatments. Undergoing gender-affirmation procedures might similarly be conceived of as a model transformative experience, but considering that, in distinction to the potentially transformative nature of PAP, in these cases, transformative experience is reflectively and actively *sought*: Conceiving and bearing a child, or the development of secondary sex characteristics of the sex with which the individual identifies, is the *aim* of these procedures.^12^ For these patients to *not* undergo a transformative experience would lead to a sense that the treatment has failed and a comparison of themselves with prospective PAP patients who suffer from depression, addiction, or another psychopathology: These patients would judge treatment as successful if their symptoms abated, whether or not they underwent a transformative experience (indeed, they may be considering other, non-transformative interventions alongside PAP).^13^ Here, the transformative experience is a likely foreseen consequence, rather than the aimed-for outcome of intervention. Although such a distinction is typically viewed as morally pertinent (McIntyre, 2019), it does not need to be accepted for how I propose we deal with the challenge of transformative experience below: much of what I recommend might equally apply to these similar procedures.

## 6. Psychedelic experiences as a challenge for informed consent

The evidence of psychedelic-induced value, behavior, and personality change remains preliminary—as do many of the findings of the psychedelic “renaissance,” but their recurrence across different treatment indications, and across different research centers, as well as how consequential these changes can be, provides sufficient cause to take seriously the possibility of such changes. This is of substantial relevance to clinical ethics: recall that a patient needs to understand the material implications of a potential treatment to provide informed consent, because this understanding is required to not only autonomously authorize an intervention but also to judge whether the treatment is right *for them*—i.e., whether undergoing the treatment is most likely to bring about an outcome that coheres with their values and preferences, but, since the relevant information about PAP is epistemically inaccessible at the point of deciding whether to commence with treatment, a patient cannot provide *informed* consent to the transformative facets of PAP as we standardly deploy the term—it must always involve a significant leap of faith: not just about what it will be like to experience PAP, but about who they might be following it.

A possible objection is that the bar for *informed consent* is being set too high here. Perhaps being informed that there is an epistemically inaccessible aspect of the treatment, that is ineffable, experientially mysterious, or personally transformative, should count as sufficiently informed for informed consent. To this, I would propose that a threshold of material understanding needs to be met for informed consent, or it does not. A lack of understanding of the material facts of another treatment would not be an acceptable basis on which to proceed with treatment. Excusing PAP from this requirement might fairly be charged with the accusation of “psychedelic exceptionalism”—“believ[ing] that the nature of the experiences people have on psychedelics are so sacred or important that the normal rules do not apply” (Johnson, 2020, p. 580). However, what is at hand here is not a special pleading to excuse psychedelics from the normal rules *because they are psychedelic*, rather, to reassess the appropriateness of the normal rules for psychedelics *because they are transformative*, along with any other medical interventions which involve transformative experience. Writing on PAP with transformative experience in mind, Smith and Sisti (2021) note that “we regularly accept consent to various activities that we cannot be fully imagined—including beginning new relationships, getting married, starting a job, and moving.” Certainly this much is true, but such experiences tend not to take place within the context of an asymmetrical, professionalized relationship between a fiduciary and a vulnerable person, governed by a duty of care. In choosing to marry someone, for example, you are making a “commitment to discover a future life together” (Paul, 2014, p. 97), a reality that is typically brought out in the rituals of the marriage ceremony. We *do* accept consent when people choose to undergo transformative experiences such as marriage or parenthood, but this is a markedly different animal to the *informed* consent that typifies clinical practice.

At this point, it would be useful to rearticulate the two functions of informed consent in clinical practice that were outlined above. The first is that of *autonomous authorization*, or ensuring *valid consent*—that is, ensuring physicians do not proceed with treatment without permission, thereby violating the rights of patients. In other contexts, *autonomous authorization* can happen on very little understanding—you can autonomously choose to gamble, even where the odds, or indeed the outcomes are not known. You can agree to pay $20 to roll a dice: roll a 1 and you'll get a surprise! Supposing you don't know how many sides the dice has, or have the faintest idea what the surprise may be, such a gamble clogs up our processes of decision-making under uncertainty at least partially how transformative experiences do.^14^ Agreeing to roll the dice is perhaps unwise. It may be liable to produce poor outcomes. However, it would be hard to call the choice *non-autonomous*, even though we know such a gambler does not understand what they are getting themselves in for. However, a higher threshold is demanded in biomedical contexts: While we do not consider a roulette player's consenting invalid when they are under the misapprehension of the “gambler's fallacy” (the last four spins were red, the next is sure to be black!), we *do* question the validity of the consent of a prospective clinical trial participant reasoning under the “therapeutic misconception” (not understanding that their trial participation is aimed at generating scientifically valid data, rather than their medical best interests).

Smith and Sisti (2021) and Smith and Appelbaum (2022), in papers reflecting more widely on ethical issues in PAP, acknowledge that there is more we could do to narrow the apparent informational gap when seeking consent. They observe that “for anyone to imagine what they would be like if their values changed or their awareness was altered is a daunting task” (Smith and Appelbaum, 2022, p. 2), arguing that the “novel risks [of PAP...] warrant an enhanced informed consent process—one that is more comprehensive than what may be typical for other psychiatric medications” (Smith and Sisti, 2021, p. 1). Smith and Sisti propose discussion prompts for the enhanced consent process (e.g., “you may feel a sense that you have lost yourself, that everything is somehow connected, or that all is one”; “you may feel a deeper connection with nature”; “you may become more spiritual—whether or not you currently consider yourself spiritual”), while Smith and Appelbaum propose facilitating exchanges between prospective patients and those who have previously been through the treatment. A prospective patient who has gone through these enhanced processes is clearly better informed than one who has not, and as such, both of these steps are valuable inclusions to the processes before treatment begins. However, if the characterization of PAP as involving transformative experience is correct, there remains an *epistemic inaccessibility* from the point of view of the prospective patient. Speaking to former patients is akin to speaking to those who have become parents to find out *what it is really like* to become a parent or speaking to a Carthusian monk to find out *what it is really like* to commit to near-total silence for a lifetime. Uncertainty can be reduced, but the perspectival shifts that result from transformative experiences can *only* come from the experience itself (Lyreskog and McKeown, 2022, p. 52).^15^

I take both of the above proposals as valuable inclusions to the processes before treatment inception: A prospective patient who has gone through these enhanced processes is better informed than one who has not. Indeed, I would argue that such patients tend to be informed enough to pass the higher threshold of understanding required for *autonomous authorization* in healthcare settings.

Although, considering that we expect consent in the medical sphere—*informed* consent—requires more than autonomous authorization, we also expect physicians, in their position as fiduciaries for their patients, to promote value-aligned decision-making by securing an understanding and rational weighing of the informative and emotional content of their decisions. However, where potentially transformative choices are at hand, comprising both an epistemic inaccessibility of the lived experience of one option, as well as a psychological incommensurability between patients before and after the treatment, such rational weighing to secure value-aligned decision-making is not possible. Recall the psilocybin for tobacco cessation study: participants who were, after many years of failed attempts, so motivated to quit smoking that they volunteered for an experimental medical trial. For some, following the trial, the changes they experienced were such that quitting smoking was of secondary importance to them.

Exposing oneself to the possibility of transformative change clearly *is* something that can be consented to, given the recognized legitimacy of the marriage ceremony. A decision to marry can (indeed, legally must) be made autonomously, even if the material, lived consequences of this decision cannot be foreseen or explained in advance. A decision to explore the unknown, to risk becoming a heretofore unknown self, can be consented to, but it is not rightly understood as *informed consent* as the term is used in clinical ethics.

## 7. Can PAP be done ethically, if transformative experience renders informed consent impossible?

Given the centrality of informed consent to modern discourse in biomedical ethics, my summative claim so far—that, when considering its potentially transformative nature, what we typically conceive of as informed consent cannot be secured for PAP—merits some exploration of whether PAP can be performed ethically. In the section, I present some considerations that can inform reflection on this question.

First, if psychedelic experiences can be transformative in nature, it may be that their transformational potential does not weigh equally heavily on all prospective patients. Certainly, the *epistemically* transformative nature of psychedelic experience is less of a concern for patients who are not psychedelically naive. Although *prima facie*, there is no reason why psychedelic-familiar patients would be immune to the potentially personally transformative effects of PAP, robust evidence on this matter is currently unavailable. Moreover, practically speaking, this does not offer much of an ethical backdoor to offering PAP without jeopardizing high standards of informed consent: Restricting PAP to those with prior experience of psychedelics would generate the perverse incentive of encouraging prospective patients to seek psychedelic experiences outside of controlled settings, or simply to lie in their medical histories, in order to access treatment.

For the physician that accepts to some degree the force of the challenge from transformative experience, drawing in broader considerations could guide decision-making. They might point to the currently limited evidence of efficacy for PAP—it has still yet to pass clinical trials—or acknowledge the methodological issues that complicate confidence in the usefulness of that data (Muthukumaraswamy et al., 2021). But this is a strategy to avoid, rather than engage with the issue—and a strategy that is unlikely to work in perpetuity. A related strategy might be to point to the state of the *comparative* evidence for PAP. Suppose that the results of the only published study directly comparing PAP to treatment as usual (Carhart-Harris et al., 2021)—i.e., no significant differences in antidepressant effects—were ultimately replicated across some or all clinical indications, in this case, the potential for infringement on informed consent might serve as justification to avoid PAP, or to leave it as a 3rd or 4th line treatment.

Another avenue could be worth exploring if the distinction I drew between PAP and other elective interventions with transformative potential (e.g., IVF and gender-affirmation procedures) is not as clear as I suggested. Previously, I suggested that IVF and gender-affirmation surgeries involve transformative change as the directly desired outcome of intervention, while this is not the case for those seeking PAP. But an argument could be made that this is not always the case for PAP. Some clinical indications for which PAP seems promising—for example severe, treatment-resistant depression, and existential distress secondary to life-threatening illness—might be framed as *treated* by a transformation of values, worldview, and priorities, in a sense that is not true for all potential applications.^16^ If this is the case, at least some uses of PAP could be as permissible as those transformative treatments.

My sense is that a better approach would be to think more deeply about what informed consent is, and why we care about it. Despite the central importance of *autonomous authorization* and *promoting value-aligned decision-making*, informed consent is not the only tool that is deployed in legitimizing medical intervention. When informed consent is not secured because it is impossible to secure, as in some emergency settings or when a patient is incapacitated, treatment is accepted as legitimate through the “emergency exception” guided by the “reasonable patient” doctrine, or through proxy decision-making (Vojta and Brown, 2015; Wrigley, 2018). Though these alternative legitimate routes are conceived of as stand-ins for informed consent, they are not informed consent *per se*. As alternative methods of treatment legitimation are acceptable in other medical contexts, it is at least in principle possible that the same can be true of PAP.

## 8. If not informed consent, then what?

Hopefully by this point, my claim—that a patient cannot provide informed consent, as we typically understand the term, to PAP—appears more plausible. It is not that a patient cannot *autonomously authorize* an intervention that involves a transformative experience. Rather, the physician's duty to support value-aligned decision-making cannot be met, if not because of the *epistemic inaccessibility* of some of the materially relevant facets of the treatment, then because of the non-clinical changes to values and priorities that can occur as a consequence of treatment. This is not to claim that physicians who currently administer PAP in the context of clinical trials, or who will do so therapeutically after licensing, are morally failing their patients in failing to secure informed consent. To the extent that PAP is transformative, informed consent is not *possible*, and therefore cannot be *required*, before beginning.

This does not need to be a problem. As outlined above, central though informed consent may be in contemporary medicine, it is not the *sine qua non* of ethical practice. It is a tool that was developed at a specific, historically contingent point, originally to minimize some of the risks associated with the asymmetrical nature of the relationship between patient and doctor—the risks of undermining autonomy, and of coercion, deception, and manipulation. Any of these charges might be leveled at a physician who intentionally withholds or misrepresents material information concerning the consequences of a treatment, including if the information is not shared in such a manner as the patient *understands* it, but in the case of transformative experience, the threshold for *understanding* cannot be met. Practitioners involved in current trials of PAP are not intentionally or negligently withholding materially relevant information—i.e., they are not deceiving or subtly coercing patients—and so the consent secured is *valid* (Bullock, 2018), but patients cannot be properly understood as *informed* about the consequences of the treatment as we usually understand it, given the forward-looking inaccessibility of the material features of the treatment. To choose PAP is, in an important respect, to choose to make a leap of faith. As considering transformative experiences in other contexts demonstrates that leaps of faith can be made autonomously, if not from a position of materially complete understanding.

What does this mean? Obviously, it does not mean that we should weaken patient protections in PAP. The increased vulnerability and suggestibility experienced during acute drug effects predispose patients to heightened risks, including the grievous harms that have come to light in recent years (Hall, 2021) and the range of other ethical challenges that arise in a modality that heightens existing relational risks of therapeutic encounters, as well as introducing new ones (Brennan et al., 2021). Rather, it demands recognition that our standard conception of *informed consent* as a legitimizing procedure in medicine may not be appropriate for PAP.

Thinking along these lines introduces another dimension along which to consider a reconceptualization of the nature of the relationship between a PAP practitioner and their patient, which is not best understood as that identical to that which typically pertains between a physician and patient. The co-discovery that takes place is more akin to that between a psychotherapist and her client (see Nayak and Johnson, 2021). Indeed, readers with less biomedical orientations may have been unperturbed by the foregoing argument on the basis that, to some approximation, the same problem from transformative experience applies to all insight-oriented (rather than symptom-focused) psychotherapies and psychoanalysis (Saks and Golshan, 2013; Poppe, 2019).

Previous writers on informed consent in long-term psychotherapies have acknowledged that “neither party knows at the outset in what directions the therapy might evolve, what information or understanding may unexpectedly emerge... or what the final outcome will be” (Beahrs and Gutheil, 2001, p. 6), but ultimately conclude that informed consent is possible even if “clear and probable outcomes cannot realistically be stated” in advance (p. 6). This position is buttressed by the temporally extended nature of long-term psychotherapeutic or psychoanalytic work, permitting the progressive, longitudinal disclosure of the material facets of the process as they arise, and affording the opportunity to discontinue treatment. In comparison, the temporally compressed nature of PAP—changes arise very quickly following psychedelic intervention—may not permit such discontinuation. Additionally, I submit that optimism about the possibility of fully informed consent to, say, psychoanalysis fails to recognize the force of the challenge from transformative experience. The issue is not simply that the possible outcomes are unknown at the outset of treatment and so cannot have likelihoods attached to them. Rather, even if the possible outcomes could be identified, a prospective patient could not know how to assign values to her options and choose between them, given that the process of coming to know those outcomes could radically alter her values and preferences in relation to them. Reflecting on some of the relational risks that can arise in psychoanalysis, Saks and Golshan write: “No one can understand something like transference or regression until it happens. And once it happens, one is not free to truly consent or decline: one is already too caught in the transference or regression to be able to escape… [Like a religious conversion,] one may imagine that if one doesn't care for it, one can just return to how one was. But arguably, after the conversion, one doesn't want to be any different. It is too late to go back” (Saks and Golshan, 2013, p. 37; see also Poppe, 2019).

While a range of safety considerations (as well as standard clinical trials requirements) demand that a lengthy consenting process be undertaken with participants in current clinical trials of PAP (Johnson et al., 2008; Smith and Sisti, 2021), the challenge from transformative experience suggests that the inappropriateness of the term “informed consent” for covering some aspects of treatment is acknowledged, both among practitioners and also with patients. Involving as it does the prescription of a controlled drug, PAP may predominantly be institutionally contained within the medical establishment when it is licensed. Nonetheless, the foregoing arguments suggest that the choice to proceed is distinct from other medical treatments, involving a process of discovery which cannot be understood before undertaken: not just about what outcomes and side-effects might arise, but what kind of values might I have? What kind of person might I become?

A modest step toward acknowledging that legitimizing procedures for PAP differ from the norm can be taken by reflecting on the etymology of *informed consent*, a term in which both words might be viewed as hangovers from a more paternalistic era of medicine (Wise, 2007). *In* + *formare* means to shape, form into, or fashion, while *com* +*sentire* is to think or feel together, to be of one mind—together implicitly characterizing a prospective patient as a passive recipient to be led to agree with a physician's better judgment about what is in their best medical interests. Changing how we describe the consenting process to include more pro-active or agential terminology—reflective commitment, decision, or choice are some options—would be a place to start, not so much because of some occult power of etymology, as because word choice can impact cognitive processes and frames of thought by invoking different mental schemata (Loftus and Zanni, 1975; Farrow et al., 2018). As well as explicitly drawing a distinction from informed consent as it is practiced for non-transformative treatments, a suitable shift in vocabulary can underline the depth of the individual nature of the decision to be made. If the doctrine of informed consent that partially grounds the physician's duty of care is poorly suited to the transformative context of PAP, what ought to replace it? *Consent*, as *autonomous authorization*, remains important, since the wrongdoing of administering PAP to an unwilling patient clearly dwarfs the challenge that comes from the epistemic inaccessibility of transformative experiences, as does the exploitation of a patient in the heightened state of vulnerability and suggestibility during acute drug effects.^17^ Similarly, PAP practitioners would be committing a serious transgression if they were to intentionally misrepresent the nature of the treatment, e.g., by overpromising with regard to its efficacy (Rucker and Young, 2021). Such risks could still be minimized by retaining the concept of *valid* consent (Cave, 2021), wherein patients are confirmed in their understanding of the broad nature of the treatment. The requirements for disclosure ought to at least include those recommended in Smith and Sisti (2021) “enhanced consent,” including an acknowledgment of the potential long-term changes in outlook, but it would be remiss of practitioners not to explicitly underline both the gravity, and the strangeness, *qua* medical intervention, of the treatment patients are about to embark upon, as contrasted both to their previous experience of medical treatments, and against the rest of their life. Patients should be made aware that there is good reason to think that the treatment they are about to undergo may be one of the most meaningful experiences of their life and that this is not universally experienced positively. Moreover, for at least some treatment indications, patients ought to know that the health condition that brought them to the clinic may, in retrospect, be of comparatively minor importance to the other dimensions of the experience. In reality, weaving these considerations into practice may not be a trivial undertaking. The information to be reviewed during the consenting procedure, relating to both the risks and realities of the acute drug experience, as well as the potentially transformative nature of the treatment in the long term, constitutes just one of the myriad factors for clinicians to consider when preparing patients for PAP. This entire process has a formative effect on the patient's “set and setting,” the extrapharmacological factors that are understood to impact the psychedelic experience itself and the outcomes that follow (Haijen et al., 2018).

## 9. Conclusion

The rebirth of PAP as a clinical tool is itself characterized by a process of discovery, being undertaken by the breadth of its supporters and stakeholders, as we attempt to find frameworks to make sense of the PAP model, especially where our standard operating procedures seem insufficient. Just as this process seeks to determine and optimize the mechanisms of action of PAP (Hendricks, 2018; Walsh and Thiessen, 2018; Fischman, 2019) and design models that might be employed to equitably provide it for those who can benefit from it (Noorani, 2020; Zelner, 2020), it will also involve interrogating the ethical frameworks supporting the treatment itself. This process of discovery will need to explore the practitioner–patient dynamic,^18^ drawing from other models of care, especially if “psychedelic therapy is like putting a magnifying glass on many of the [relational] aspects of non-psychedelic therapy,” wherein the practitioner is “associated with what might be one of the [most] meaningful experiences in a person's life” (Johnson, 2020).

Does the foregoing—suggesting that informed consent is not an appropriate norm for addressing the potentially transformative nature of PAP—amount to “psychedelic exceptionalism”? To answer this, I will draw a parallel to the scientific challenge for PAP that began the current study, on the appropriateness of the double-blind randomized placebo-controlled trial. To bypass the drug evaluation processes for PAP in their entirety—as in the successful Oregon ballot initiative to legalize “psilocybin services”—is clearly to excuse psychedelics from “the normal rules” of drug safety and efficacy testing. However, consider that PAP is hypothesized to partially depend on psychotherapeutic support and involves hard-to-blind subjective effects, not as bugs but as features. To insist that its efficacy testing wholly conform to rules that are designed to discount extrapharmacological factors, and which depend on neither patient nor practitioner knowing if an active agent is at work, is to focus on the rules rather than what the rules seek to secure. While pragmatic and feasible steps can be made to conform PAP trial processes to better fit established norms (Aday et al., 2022), we should remain flexible to the reality that the valued end at which these trials aim—objective drug efficacy evaluation—can also be approached by incorporating complementary evaluation methodologies, without compromising our epistemic standards (Butler et al., 2022). In a similar vein, if the transformative nature of PAP is such that informed consent, as we standardly understand it, is not feasible, pragmatic steps including those suggested by Smith and Sisti (2021) and Smith and Appelbaum (2022) can narrow the gap between current practice and the normative ideal, but if this much—as I have suggested—is not enough to completely conform to “the normal rules,” we should not lose sight of the end at which those rules aim, namely, ethical treatment of patients.

The gold-standard status of the double-blind placebo-controlled trial is akin to the prized position of informed consent in clinical ethics, but neither is manna from heaven: They developed at specific, historically contingent points in time to meet the perceived needs of the moment (Faden and Beauchamp, 1986; Berg et al., 2001, Ch. 14; Oram, 2018). Acknowledging that our most powerful tools may not be the best approach to solving all problems—that they may not always live up to our expectations for them—does not lessen their general value (Berg et al., 2001). As new medical techniques and technologies develop, the more likely it is that our medical ethics must be revised to keep in step (Einav and Ranzani, 2020).

## Data availability statement

The original contributions presented in the study are included in the article/supplementary material, further inquiries can be directed to the corresponding author.

## Author contributions

The author confirms being the sole contributor of this work and has approved it for publication.

## Footnotes

^1^For example, to the extent that a valid consent to a surgical procedure under anesthetic permits an action that would otherwise be an assault on the right to bodily integrity, such a consent does not constitute carte blanche against any violation of bodily integrity, be it sexual touching or the opportunistic removal of an appendix that was found to be infected during surgery.

^2^That we place value on treatment decisions being value-aligned or *authentic* to the patient in this way is perhaps most clearly seen in a context where informed consent is not possible: In cases of surrogate decision-making, when needing to make a decision for a temporarily incompetent relative, we naturally ask “what would they choose” (Brudney, 2009).

^3^With thanks to a reviewer for presenting at this juncture the example of a medical abortion chosen by someone who considers abortion morally wrong. This example neatly demonstrates that informed consent does not require that the chosen procedures must align with patient values, rather, that patients are made aware of how treatment options interact with their values.

^4^It is noteworthy that some writers conceive of this function of informed consent as a duty both to doctors *and* patients: “Doctors are obligated to facilitate patients' opportunities for reflection to prevent ill-considered rational and irrational influences on choice. Patients, in turn, are obligated to participate in the process of thinking about choices” (Katz, 2002, p. 122).

^5^As in, for example, accounts that set the threshold for the material aspects of a treatment that must be disclosed as that which the physician judges to be material to the patient or that which the patient judges to be material.

^6^On this rendering, a good many “first-time” experiences are epistemically transformative, even trying a new fruit for the first time, but note that the experience of having one's first child is a radical and profoundly consequential departure from a person's previous experience, along multiple dimensions, compared to having one's first guava.

^7^As Paul frames this double dilemma when trying to weigh whether undergoing a transformative experience will be right for us, “[w]e only learn what we need to know after we've done it, and we change ourselves in the process of doing it” (Paul, 2014, p. 4).

^8^Cf. Lyreskog and McKeown's (2022, p. 51) description of a transformative experience as “one with such a profound impact that once having had it, one would recognize the testimony or observation of others who had it as falling far short of communicating what the experience is like.”

^9^Patient reports of synesthetic experiences from Belser et al. (2017) include “my entire body was musical instrument for every sound, which was coming through my head, and it eviscerated from top to bottom... I know what a grand piano feels like when it is played.” “I started tasting music... some of Indian instruments have very sharp chords, those felt metallic in my mouth.”

^10^This is not necessarily the case for prospective patients who are not psychedelic naïve (about whom see more in Section 7 below), although my sense, arguably supported by the existence of subjective phenomenological scales with strong discriminant validities between different pharmacologically-induced altered states of consciousness (Studerus et al., 2010), is that these experiences are significantly novel even for those familiar with other drug-induced intoxications. Additionally, there may be some prospective patients who have had sufficiently similar experience from advanced meditative practice, or holotropic breath work, or congenital synesthetic perceptual processing. Regardless, the radical novelty of the psychedelic experience will likely hold true for most patients.

^11^With thanks to a reviewer for this highly consequential observation.

^12^The same may be true of perhaps the ultimate transformative experience that is the goal of medical assistance in dying.

^13^Here, I restrict myself to medical applications of psychedelics, rather than the expressly non-medical uses as permitted by, e.g., recent legislational changes in Oregon, for which distinct consenting frameworks are appropriate.

^14^Undertaking such a gamble is, after all, *epistemically transformative*, if not *personally transformative*.

^15^On the potential and the shortcomings of clearing the “epistemic wall” through testimony, literature, and imagination, see Ismael (2019), Paul (2019), and Woollard (2021).

^16^Although this may depend on the nature of the broader psychotherapeutic programme in which the drug sessions are contained (Devenot et al., 2022).

^17^Indeed, some underground practitioners *double up* on the use of consent during acute drug effects in recognition of the special risk to autonomy that they pose, using a two-stage consent process to touch, seeking consent both before psychedelic administration and again during acute drug effects (Brennan et al., 2021).

^18^See Timmermann et al. (2020) for one such example.
